# Synergistic effects of vedolizumab and JAK 1,2,3 inhibitors in Crohn’s disease: insights from a systems biology and artificial intelligence-based approach

**DOI:** 10.3389/fimmu.2025.1699203

**Published:** 2025-12-10

**Authors:** Ignacio Marín-Jiménez, Mónica Sierra-Ausín, Teresa Letosa-Abián, Jesús Aparicio, Carmen Montoto-Otero, Silvia Sánchez-Ramón

**Affiliations:** 1 Servicio de Aparato Digestivo, Unidad de Enfermedad Inflamatoria Intestinal, Centro de Enfermedades Inmunomediadas (CEIMI), Hospital General Universitario Gregorio Marañón, Instituto de Investigación Sanitaria Gregorio Marañón (IiSGM), Universidad Complutense de Madrid, Madrid, Spain; 2 Unidad de Enfermedad Inflamatoria Intestinal, Servicio de Aparato Digestivo, Complejo Asistencial Universitario de León, León, Spain; 3 Takeda, Medical Department, Madrid, Spain; 4 Departamento de Inmunología Clínica, Instituto de Medicina de Laboratorio y IdISSC, Hospital Clínico San Carlos, Madrid, Spain; 5 Departamento de Inmunología, Oftalmología y ORL, Facultad de Medicina, Universidad Complutense de Madrid, Madrid, Spain

**Keywords:** vedolizumab, JAK 1,2,3 inhibitors, systems biology, Crohn’s disease, artificial intelligence, advanced combination therapy

## Abstract

**Background and aims:**

While current therapies for Crohn’s disease (CD), including vedolizumab (VDZ) and Janus kinase inhibitors (JAKi), have shown efficacy individually through different mechanisms of action, their combination may offer a more integrated approach, potentially improving outcomes for refractory or severe CD patients. By leveraging *in silico* systems biology and artificial intelligence, we provide a valuable method to understand the molecular mechanisms underlying this combined therapy.

**Methods:**

CD, VDZ, and JAKi were characterised at protein level by compiling data from extensive literature review and official documents. CD protein effectors were identified and used to construct mathematical models using Therapeutic Performance Mapping System (TPMS) technology. Sampling-based methods were employed to assess the impact of the combined VDZ plus JAKi therapy on CD and to unveil underlying molecular mechanisms.

**Results:**

Disease characterisation identified 4 pathophysiological processes (referred to as motives, M) underlying the manifestation of the disease: intestinal barrier disruption (M1), altered innate immune response (M2), chronic inflammation and predominant Th1/Th17 adaptive immune response (M3), and tissue remodelling (M4). The combination of VDZ and JAKi modulated more effectors of CD (54.8%) than each drug did individually, thereby enhancing the effects of each drug. The combination therapy modulated 45 effectors (36%) through convergent mechanisms, primarily impacting M3, and also provided complementary mechanisms across all motives, but mainly in M1 and M4.

**Conclusions:**

Our results provide a mechanistic rationale for combining VDZ and JAKi as a potential therapy for CD, advancing the development of more effective and personalised treatment strategies.

## 1 Introduction

Inflammatory bowel disease (IBD) represents a spectrum of chronic inflammatory gastrointestinal disorders, notably including Crohn’s disease (CD) and ulcerative colitis (UC), wherein active disease alternates with variable periods of remission (1, 2). These conditions manifest through diverse clinical symptoms, including abdominal pain, diarrhoea, bloody stools, urgency, fatigue, weight loss, as well as extraintestinal manifestations. These symptoms are mainly attributed to the infiltration of neutrophils and macrophages, leading to inflammation and ulceration within the digestive tract (1–3). Specifically, CD is characterised by patchy transmural inflammation and mucosal lesions that can affect any part of the gastrointestinal track (2). The incidence and prevalence of IBD have shown a significant increase globally over recent decades, now affecting up to 1 in 200 individuals in Western countries (1, 4, 5). In Spain, a recent a population-based nationwide study yielded an overall incidence of 7.5 cases/100,000 person-years for CD (6). Understanding the aetiology of CD necessitates recognizing its complex, multifactorial nature. The interplay among the three main factors –genetic susceptibility, gut immune response, and microbiota dysbiosis– is influenced by environmental triggers, ultimately contributing to disease development (1, 2, 7).

Therapeutic strategies for CD have evolved over time, with both non-biological and biological therapies being employed. However, these approaches have shown limited efficacy, loss of response, and adverse events (3, 8–11). Advanced treatments report clinical remission rates at 1 year up to a maximum of 50% (12) suggesting that a therapeutic ceiling has been reached using single agents (12–14). The efficacy plateau observed with advanced monotherapies underscores the growing interest in innovative treatment modalities, such as combination therapies. Taken together, clinical and preclinical data suggest that combination approach might potentially break through that efficacy ceiling (12, 14), although data from controlled studies are still scarce (13, 15).

Vedolizumab (VDZ) is a humanised monoclonal antibody targeting α4β7 integrin expressed on circulating lymphocytes, selectively preventing their migration into gut mucosa (16, 17).The α4β7 integrin is preferentially expressed by a subset of CD4^+^ memory T cells and mediates gut-selective homing. Memory T cells expressing α4β7 migrate selectively into the gastrointestinal tract by binding to the mucosal addressing cell adhesion molecule-1 (MAdCAM-1) (8). α4β7 is also expressed by dendritic cells (DCs), CD8^+^ T cells, B cells, plasmablasts, innate lymphoid cells, basophils, and eosinophils, playing an important role in trafficking of immune cells into the intestinal mucosa (18). VDZ binds exclusively to the gut-tropic α4β7 integrin, avoiding systemic side effects reported with other anti-integrin monoclonal antibodies, such as natalizumab (8). Thus, VDZ is recognized as an effective therapeutic option to treat moderate to severe CD (8, 19). Additionally, targeting cytokines involved in the pathology of the disease through Janus kinase inhibitors (JAKi) has shown promise in the treatment of CD. Janus kinases, including JAK1, JAK2, JAK3, and TYK2, comprise a mammalian family of intracellular enzymes involved in signalling pathways that affect haematopoiesis and immune cell function (20, 21). JAKi, including tofacitinib, filgotinib and upadacitinib, have been approved for UC, and to date, upadacitinib is the only JAKi approved for patients with moderate to severe CD (20–22). There are still few data on the combination treatment of VDZ plus JAKi, although there are some clinical evidences of the benefits of such treatment for IBD in refractory and selected patients (9). Combining VDZ with JAKi presents a potential strategy to overcome primary non-response or secondary loss of response by simultaneously targeting differential disease-mediating mechanisms (3, 23). Preliminary published results have shown that the combination of VDZ plus JAKi tofacitinib is effective in UC patients without significant safety concerns (23) More recently, a retrospective observational study has reported favourable clinical and endoscopic outcomes and safety profile with the combination of VDZ with Upadacitinib in patients with refractory CD and UC (24). Nevertheless, the underlying molecular mechanisms responsible for the improved outcomes in UC patients treated with VDZ plus JAKi remain unknown, and whether this combination is effective in CD patients still needs to be fully confirmed.

In the pursuit of a deeper understanding of drug mechanisms of action (MoAs) and disease pathophysiology, systems biology and artificial intelligence (AI) offer valuable tools (25). In this context, Therapeutic Performance Mapping Systems (TPMS) has emerged as an effective technology that simulates the physiological effects of pharmacological compounds or biological processes at the molecular level, providing insights into the molecular MoAs associated with a given drug and bridging the molecular and clinical worlds (25). Its applicability has already been proven across several therapeutic areas (26–29), including combined therapies (30).

In order to obtain a holistic and mechanistic understanding of the potential benefits of combining VDZ and JAKi in CD, in the present study we employed TPMS technology to reveal MoAs at the molecular level that can potentially explain how these drugs interact to modulate disease pathways and improve patient outcomes. By gaining a better understanding of VDZ and JAKi MoAs in CD, researchers can potentially identify more specific therapeutic targets and improve treatment outcomes for refractory or severe CD patients.

## 2 Methods

**Figure 1:**
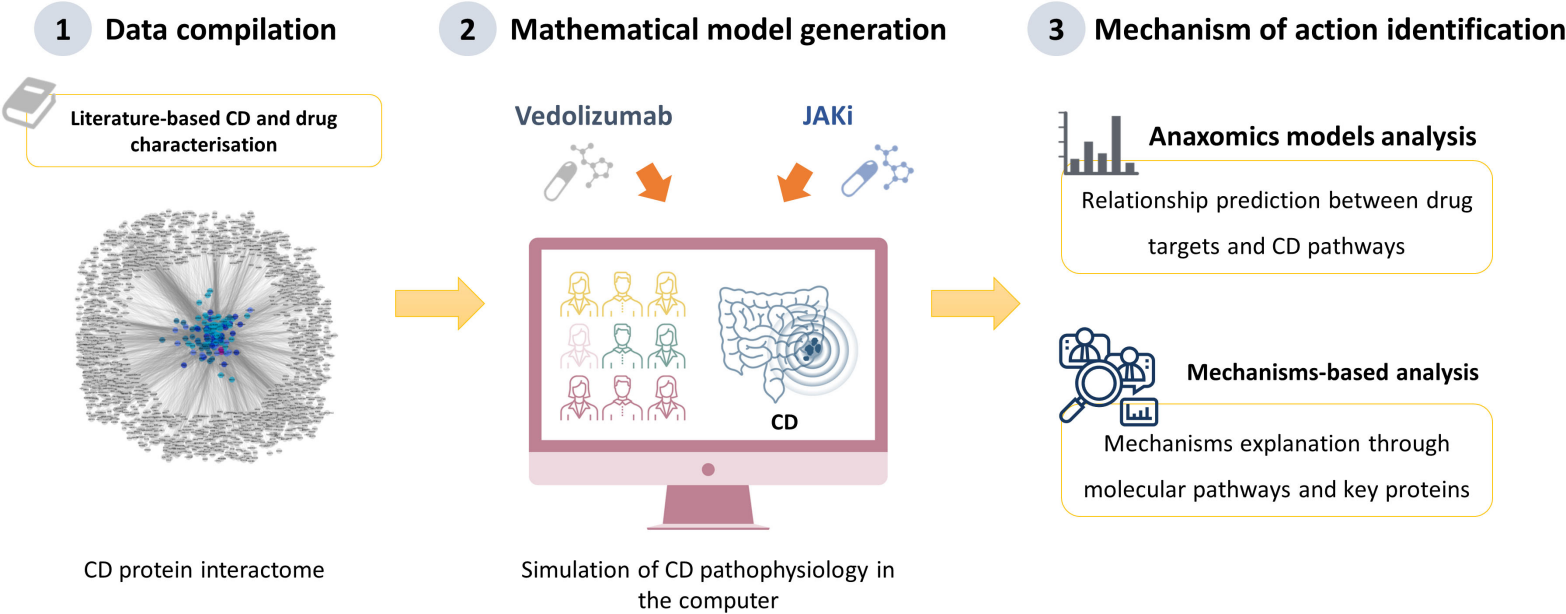
Schematic representation of the workflow and key steps in the project. The figure represents the Therapeutic Performance Mapping Systems (TPMS) approach to analyse the mechanisms of action (MoA) of vedolizumab (VDZ) plus JAK inhibitors (JAKi) in Crohn’s disease (CD). TPMS is based on systems biology-based models and encompasses three steps **(1)**: molecular characterisations of the disease and drugs through a comprehensive bibliographical revision, from which CD interactome can be constructed using the protein-protein interaction (PPI) human network **(2)**; the learning process of the PPI human network, based on training and validation with known information stored in the training set; this learning is performed with machine learning techniques to construct accurate mathematical models that simulate the behaviour of human physiology; and **(3)** analysis of specific MoA models for the impact of drug treatment on CD [Jorba 2020; Segú-Vergés 2023].

TPMS developed by Anaxomics Biotech (Barcelona, Spain), represents a ground-breaking methodology at the intersection of top-down systems biology, AI, and pattern recognition models. Previous published works (25, 29, 30) outline TPMS as a powerful tool bridging the gap between molecular and clinical realms, providing intricate insights into the physiological effects of pharmacological compounds and biological processes at the molecular level. TPMS technology integrates extensive biological, pharmacological, and medical knowledge to simulate in silico normal and pathological human physiology through mathematical models. This integration allows for a detailed understanding of the molecular mechanisms underlying diseases and drug actions. Modelling the effect of a drug is crucial to understand how it interacts with disease mechanisms. In this study, we used TPMS technology to simulate the clinical responses of CD to treatments with VDZ and JAKi, providing a detailed view of the MoA and how these treatments can reverse the pathological effects of CD. **Figure 1** summarises the project’s workflow and key steps used to identify potential pharmacological mechanisms underlying the actions of VDZ in combination with JAKi in the treatment of CD patients. To corroborate the model, we used bioflags, defined as genes or proteins altered by the effect of the drug on its targets and/or off-targets at the downstream level, based on the literature. These bioflags serve as biological markers to verify that the model accurately represented the drug effects.

### 2.1 Targeted literature search for molecular characterization of VDZ and JAKi MoA and CD pathophysiology

The first step in our study was the compilation of all available information regarding CD and the drugs under study (**Figure 1**, step 1). According to the previously described methodology (26–29), the pathogenesis and pathophysiology of CD were molecularly characterised through manual curation of recent scientific literature, detailing the molecular and cellular pathways involved. This information was used to build the protein network and the mathematical model describing CD. Only English-language articles were included. The search string used in PubMed (up to March 2023) is detailed in **Supplementary Table S1**. The retrieved results were assessed at the title and abstract or full text level, depending on whether they contained molecular information. This process entailed the identification of four key pathophysiological processes (referred to as “motives”) involved in the pathophysiology of CD: 1) intestinal barrier disruption, 2) defective innate immune response, 3) chronic inflammation and Th1/Th17 adaptive immune response, and 4) tissue remodelling. Motive details are summarised in **Supplementary Table S2**. The four motives were further characterised at protein level (**Supplementary Table S3**). The publications retrieved were used to screen protein/gene candidates as condition effectors by the association of their functional activity (1) —or lack thereof (–1) — with disease development. If scientific evidence for a potential candidate was not consistent enough, an additional PubMed search was performed including all the protein names according to UniProtKB. Novel candidates identified at this step were added to the list of effectors, following the same criteria and protocol. A total of 148 unique protein effectors were identified for CD.

Similarly, drugs under study in this project (VDZ and JAK1,2,3 inhibitors: tofacitinib, upadacitinib, and filgotinib) underwent molecular characterisation, with their targets and downstream effects documented through a review of official documents, including the European Public Assessment Report (EPAR, European Medicines Agency), the Food and Drug Administration, and Product Monographs, specialised databases (DrugBank), and scientific literature in PubMed from the last 10 years. Titles and abstracts were screened, and articles containing relevant molecular information were thoroughly reviewed to identify protein or gene candidates as potential drug targets or bioflags, as well as other useful information regarding its mechanism of action in CD. **Supplementary Tables S4, S5** provide detailed description of compiled drug targets and bioflags, respectively. The search strings for VDZ and JAKi used in PubMed (up to March 2023) are detailed in **Supplementary Table S1**.

### 2.2 Generation of systems biology-based models for VDZ plus JAKi on CD

TPMS mathematical models (**Figure 1**, step 2) are built over the Human Protein Network (HPN), as previously described (25, 29). This protein-protein interaction human network incorporates the available relationships between proteins from a regularly updated in-house database drawn from public sources. In order to transform the static HPN into mathematical models capable of both reproducing existing knowledge and predicting new data, a collection of known input (drugs)-output (clinical conditions) physiological relationships was used as training data, or “training set”, to train the models. This training set was constructed using a compendium of biological, pharmacological, clinical databases, and literature through text mining techniques and manual review of the information to obtain curated, experimentally supported input–output relationships between drugs, targets, and clinical conditions (25, 29, 30). The algorithm used for the training of TPMS models is similar to a Multilayer Perceptron of an Artificial Neural Network over the HPN (where neurons are the proteins and the edges of the network are used to transfer the information) (25, 29, 30). The parameters to solve are the weights associated to the links between every node pair (ω_x_). The ω_x_ parameters are obtained by optimization, using a Stochastic Optimization Method based on Simulated Annealing (31), which uses probabilistic measures derived from the biological evidence to adjust network interaction types and strengths. The integration of the input signals over each of the nodes (proteins) of the network consists of the sum of all input values that arrive at the node. This signal is submitted to a sigmoid function to produce a normalized output in the range [-1,1] (hereinafter “Predicted protein activity”), this output-signal being the input-signal for the next node, weighted according to the link weight ω_x. The topology is initialized by random values for all the links in each of the initial models, and each model is evaluated against the training set several optimization cycles, until it stabilizes (26, 29, 30). Every mathematical model created must satisfy this training set, as previously detailed (25, 29, 30). Thus, the models are optimized to, besides propagating the signal from the stimulus to the response, reproducing every rule contained in the training set; the accuracy is then calculated as the sum of all the rules complied with. The accuracy reflects the internal consistency of the model with established pharmacological and pathophysiological knowledge (25, 29). Given that the number of *ω_x_* parameters (i.e. protein-protein interactions) is bigger than the collection of input-output relationships in the training set, a variety of valid models, or *solutions*, are obtained (25); thus, the final model is rather an “ensemble of models” that represents a universe of plausible solutions than a single, unique model. This diversity can be viewed as a reflection of natural interpatient molecular variability. The solutions with the highest accuracy against the training set are selected and considered for further analysis, considering valid only those solutions with accuracy above 90%.

TPMS technology was used to simulate in silico molecular, cellular and tissular features of CD pathophysiology, exploring all plausible relationships between an input or stimulus (drug targets) and an output or response (CD motives). This type of analysis helps to elucidate the molecular mechanisms by which drug-pathophysiology relationships function and how the treatment can modulate the pathophysiology (25, 26, 30). Specifically, three models were developed: one for VDZ treatment, one for JAKi, and one for their combination. Developing separate models allowed for a detailed evaluation of how each treatment modulates disease mechanisms, and whether their combination can offer additional benefits through synergistic effects. The combined model included integrin α4 and integrin β7 as VDZ targets, and JAK1, JAK2, JAK3, and TYK2 as JAKi targets. Due to limitations in the modelling system, MAdCAM-1 was added as a pseudotarget of VDZ to help the model better simulate the connection between the drug and its downstream effects, thereby enabling an adequate reproduction of the impact of integrin modulation on the pathophysiology of CD. Although VDZ does not directly bind to MAdCAM-1, its inhibition in the model represents the non-binding of MAdCAM-1 to integrin α4β7, as described in the literature, and allows for an equal downstream treatment of JAKi and VDZ with regards to the underlying tissue.

### 2.3 MoA identification: model evaluation and validation

Sampling-based methods were used to evaluate the MoAs and assess the impact of VDZ, JAKi, and VDZ plus JAKi on CD-simulated patients through mathematical models (**Figure 1**, step 3). The resulting models, comprising a set of solutions, are able to describe all plausible mechanistic relationships, or MoAs, between an input and an output. The defined “Predicted protein activity” between 1 and -1, indicates activation and inhibition, respectively, that each protein in the MoA subnetwork achieves can be used to obtain composite measurements for CD or each of the motives. We defined full T signal (fSignal) as the average signal that arrives at the protein effectors. The fSignal is a measure that evaluates the intensity of the signal generated by the stimulus (drug) that reaches the response set (disease). According to the TPMS methodology, a variance-based Sobol sensitivity analysis (32) is applied to quantify the contribution of individual model parameters to the overall TPMS output signal (considered as a function tSignal=TPMS(X), where Xrepresents protein activity states). Monte Carlo simulations introduced controlled perturbations to the input across the model solutions. Variance decomposition of the resulting outputs enables the identification and quantitative ranking of key proteins and pathways driving the system’s mechanistic behavior.

To elucidate the impact of the drugs on each pathophysiological motive featuring the condition, we analysed the fSignal and the percentage of effectors reverted by the drug over the disease pathophysiology, as previously described in detail (25, 30). The fSignal retrieves the signal values of all response proteins (CD effectors) after stimulating the model, that is, propagating a signal through the drugs’ targets over the network, and computes the mean value. The percentage of reversed effectors measures the ability of each treatment to reverse the protein activity status in these pathological mechanisms, according to the molecular characterisation. We defined “reverted effectors” as the proteins that are activated/inactivated by the drug in the opposite activation state than in the CD molecular characterisation, with at least |0.1| protein activity. We calculated the percentage of reversed proteins in each motive to gain a quantitative point of view of the stimulus’s impact. These proteins were considered to evaluate the contribution of each drug to the combination efficacy. However, it is not appropriate to interpret these values as a quantitative measure of efficacy. Accuracy of the final MoA models was calculated as the mean of the accuracies of all considered solutions. Once the individual mechanisms for each drug were identified, they were jointly evaluated to identify the potential of the combination in the treatment of CD. The percentage of reversed effectors measures the ability of each treatment to reverse protein activity status in the pathological mechanisms, according to the molecular characterisation.

### 2.4 Software

All simulations described in this project were conducted on Anaxomics’ cloud computing platform, which integrates over 800 computational threads in machines with 64 Gigabytes of RAM. Software, databases, and tools used are the property of Anaxomics Biotech. Graphs representing the mechanism of action were generated using Graphviz software (33).

### 2.5 Ethical statement

The present study was not subject to medical research involving human subjects or living animals. Therefore, it did not require written informed consent from patients or approval from Ethical Research Committees.

## 3 Results

### 3.1 Mechanistic model of VDZ plus JAKi on CD through TPMS

TPMS technology was used to develop mechanistic system biology-based model of the actions of VDZ plus JAKi on CD pathophysiology. A key element of model creation is to build a protein-protein interaction network based on the identified CD effectors (see **Supplementary Table S3**), which serves to place these identified effectors within the broader context of all reported human protein interactions. A comprehensive review of the literature allowed us to identify four key pathophysiological processes in CD (so-called motives, M), which are as follows: 1) intestinal barrier disruption (motive 1, M1); 2) altered innate immune response (motive 2, M2); 3) chronic inflammation and predominant Th1/Th17 adaptive immune response (motive 3, M3); and 4) tissue remodelling (motive 4, M4). These four motives represent the main underlying mechanisms in the manifestation of the disease. The molecular and functional characterisation of these motives yielded 50, 24, 45, and 59 proteins (so-called effector proteins) associated with M1, M2, M3, and M4, respectively. The total number of unique effector proteins was 148 (see **Supplementary Table S3**).

M2 represents an early pathophysiological event that triggers chronic inflammatory responses, eventually leading to CD establishment (34). However, VDZ and JAKi are approved for the treatment of moderate to severe CD, when the disease is fully established, not during the time-period when M2 occurs. In addition, a preliminary evaluation of the models for each drug showed that no signal reached M2 effectors, having no clear impact on this motive (see **Supplementary Figure S1**). Considering these factors, M2 was not included in our modelling. Nevertheless, exclusion of M2 does not imply the complete exclusion of the innate immune response from our modelling analysis, as molecular players involved in innate immunity are also represented in our modelling in motives M1, M3, and M4 (11). More specifically, our analysis showed that 14 out of the 50 effectors found in M1, 21 out of the 45 effectors in M3, and 17 out of the 50 effectors in M4 involved the innate immune response.

In conclusion, we considered M1 (intestinal barrier disruption), M3 (chronic inflammation and predominant Th1/Th17 adaptive immune response), and M4 (tissue remodelling) to model CD in this project. Additionally, the individual profiles of VDZ and JAKi protein targets were characterised (**Supplementary Table S4**). Potential biological mechanisms occurring in CD after VDZ plus JAKi treatment were described using sampling methods (see Methods section). This sampling-based methods model presented an accuracy against the pharmacological and pathophysiological human knowledge contained in the training set of 93.95%.

### 3.2 Effects of the combination therapy in the treatment of CD

The predicted MoAs for each drug were jointly evaluated, aiming to elucidate the likely pharmacological mechanisms evoked by the combination therapy on CD. The impact of each treatment on the specific biological processes (motives) involved in this disease was assessed through two complementary parameters: 1) the percentage of altered effectors reversed by the drug, and 2) the intensity of the response measured using fSignal. These parameters provide an idea of the scope and the strength of the treatment’s impact on a specific biological process.

**Figure 2:**
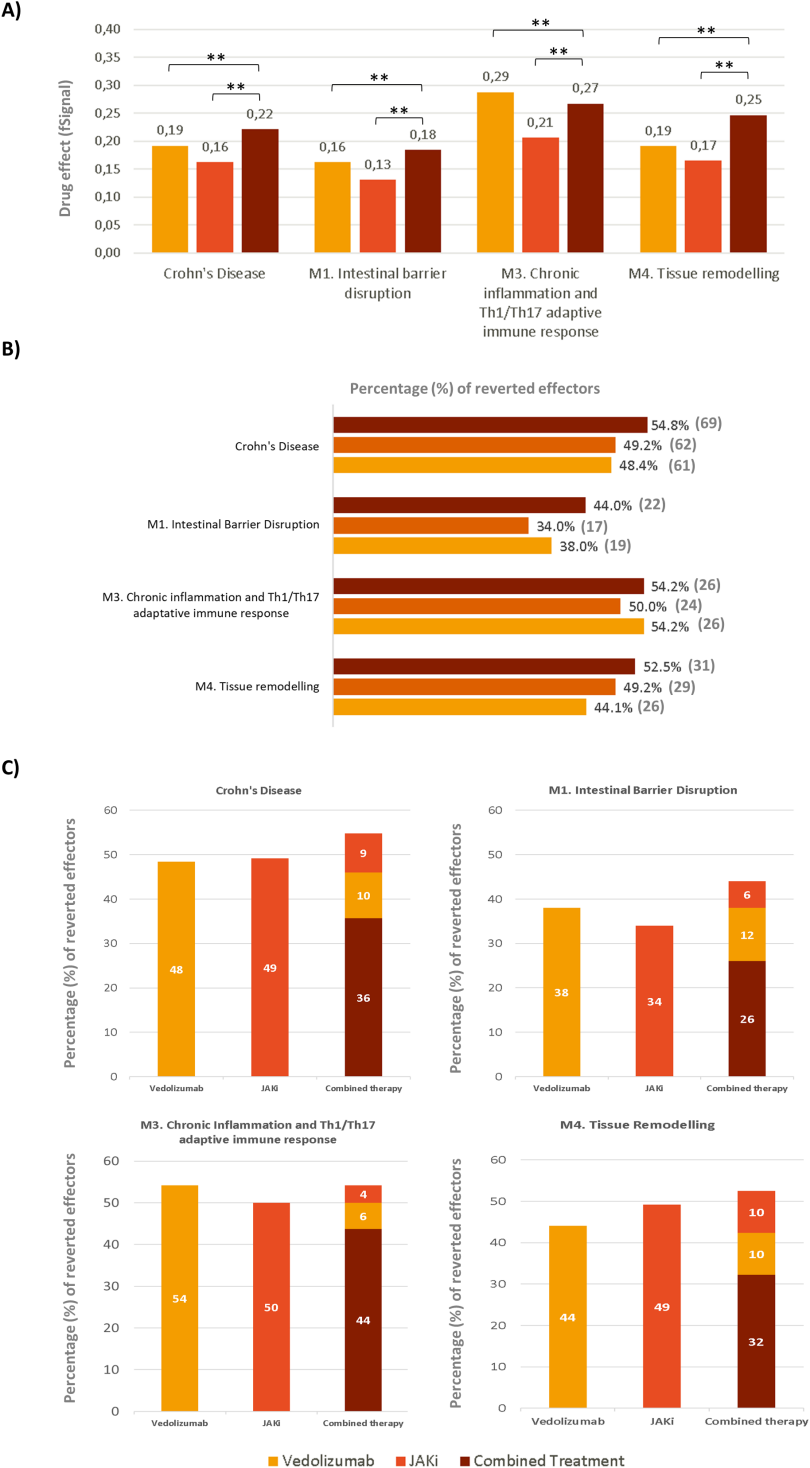
VDZ, JAKi, and VDZ plus JAKi effects versus CD and each pathophysiological motive. **(A)** Signal values (fSignal) of all response proteins (CD effectors) after stimulating the model. **p-value < 0.01. **(B)** Percentage of altered effectors reversed by each drug and the combined therapy in CD and each specific motive. Numbers in parenthesis represent number of protein effectors. **(C)** Representation of the percentage of protein effectors modulated by each single therapy and by the drug combination. Yellow bars represent vedolizumab treatment alone, orange bars represent JAKi treatment alone and brown bars represent the combination therapy.

As shown in **Figures 2A, B**, at single treatment level, the fSignal was slightly higher for VDZ than for JAKi; however, both drugs modulated a similar number of protein effectors on disease pathophysiology. More specifically, VDZ was able to revert more protein effectors in M1 and M3, while JAKi were able to revert a higher percentage of proteins effectors in M4. Therefore, the combination of VDZ plus JAKi, unlike other combined treatments, would act at different stages of the disease, thus offering potential greater benefits to CD patients. Combined treatment showed both an increased fSignal and a higher percentage of reversed effectors (54.8%) that any of the therapies individually. Specifically, the combined therapy reversed the activity of more effectors than the individual treatments in M1 and M4 and equalled the number of effectors reversed by VDZ in M3. **Figure 2C** shows that both VDZ and JAKi converged in the modulation of 45 proteins, which represents 36% of the total CD effectors. Moreover, each drug provided complementary mechanisms for the improvement of CD pathophysiology, since 10% (13 proteins) of the reversed effectors were due to the exclusive effects of VDZ, while 9% (11 proteins) were reverted by the exclusive effects of JAKi.

Our modeling analysis point out that the combination of VDZ plus JAKi primarily impacts M3, where both drugs exhibit a high degree of convergence despite their distinct MoAs. However, the complementary mechanisms of these drugs are evident across all motives, with a pronounced impact observed on M1 (intestinal barrier disruption) and M4 (tissue remodeling). This differential targeting of M1 and M4, along with the convergence on M3, potentially amplifies therapeutic coverage and implies synergistic effects (by modulating common effectors) and additive effects (by providing complementary mechanisms) between VDZ and JAKi.

### 3.3 Contribution of each drug to the combined therapy

The contribution of each drug to the overall MoAs was evaluated focusing on complementary mechanisms and convergent effectors. Complementary mechanisms describe the pathways characteristic of the individual drugs that are maintained in the combination treatment and provide additional mechanisms that complement the convergent effects of the drug. Results are summarised in **Table 1**. Specifically, VDZ exclusively reverted 13 CD effectors: 6 from M1 (NFKB2, CLDN1, CLDN2, CLDN3, OCLN and TJP1), 3 from M3 (ITGA4 and ITGB7 (drug targets) and NFKB2), and 6 from M4 (PDGFB, IL5, IL6, IGF2, MMP9 and NFKB2). Likewise, JAKi exclusively reverted 11 CD effectors: 3 from M1 (WNT1, PPARG and IL22), 3 from M3 (TLR4, MICB and IL22), and 7 from M4 (IL13, TGFBR2, EGF, COL1A1, COL1A2, MSH2 and WNT1). Thus, the combination therapy provides complementary pathways from each drug that promote mainly the reversion of M1 and M4 motives.

**Table 1:** Complementary mechanisms provided by the individual drugs to the combined treatment.

Gene name	Motive	Activation sign in disease	Vedolizumab	JAKi	Combination
ITGA4	3	↑	▼	▬	▼
ITGB7	3	↑	▼	▬	▼
PDGFB	4	↑	▼	▬	▼
NFKB2	1; 3; 4	↑	▼	▬	▼
IL5	4	↑	▼	▬	▼
IGF2	4	↑	▼	▲	▼
MMP9	4	↑	▼	▲	▼
IL6	4	↑	▼	▲	▼
CLDN2	1	↑	▼	▲	▼
TLR4	3	↓	▼	▲	▲
IL13	4	↓	▼	▲	▲
MICB	3	↑	▬	▼	▼
TGFBR2	4	↑	▬	▼	▼
WNT1	1; 4	↓	▬	▲	▲
PPARG	1	↓	▬	▲	▲
IL22	1; 3	↓	▬	▲	▲
EGF	4	↑	▲	▼	▼
COL1A1	4	↑	▲	▼	▼
MSH2	4	↑	▲	▼	▼
COL1A2	4	↑	▲	▼	▼
CLDN1	1	↓	▲	▬	▲
OCLN	1	↓	▲	▼	▲
TJP1	1	↓	▲	▼	▲
CLDN3	1	↓	▲	▼	▲

The proteins that exhibit a change in the activation status after vedolizumab or JAKi treatment, and is enhanced in the combination therapy, are represented. The corresponding motives for these proteins are indicated: intestinal barrier disruption (M1), chronic inflammation and Th1/Th17 adaptive immune response (M3), and tissue remodelling (M4). Activation sign reflects protein status as compiled in the disease characterisation. Green arrow/triangle indicates activation, red arrow/triangle indicates inactivation, and yellow hyphen indicates that the individual drug does not affect the effector protein.

Convergent effectors are defined as CD protein effectors reversed by VDZ and JAKi independently but predicted to be greatly reversed when both drugs are combined. 10 proteins were identified as convergent effectors (TCF4, IFNG, FASLG, CCR9, MMP1, CDH1, NFKB1, PLA2G1B, PAMK3 and MAPK1). Results are shown in **Table 2**.

**Table 2:** Convergent mechanisms from the individual drugs in the combined treatment.

Gene name	Motive	Activation sign in disease	Vedolizumab	JAKi	Combination
CCR9	3	↑	▼	▼	▼
CDH1	1	↓	▲	▲	▲
FASLG	3	↑	▼	▼	▼
IFNG	1, 3, 4	↑, ↑, ↓	▼	▼	▼
MAPK1	4	↑	▼	▼	▼
MAPK3	4	↑	▼	▼	▼
MMP1	1, 4	↑, ↓	▼	▼	▼
NFKB1	1, 3, 4	↑, ↑, ↑	▼	▼	▼
PLA2G1B	1	↑	▼	▼	▼
TCF4	1, 4	↓, ↑	▼	▼	▼

The proteins that exhibit a change in the activation status after vedolizumab or JAKi treatment, and is maintained in the combination therapy, are represented. The corresponding motives for these proteins are indicated: intestinal barrier disruption (M1), chronic inflammation and Th1/Th17 adaptive immune response (M3) and tissue remodelling (M4). Activation sign reflects protein status as compiled in the disease characterisation. Green arrow/triangle indicates activation and red arrow/triangle indicates inactivation.

### 3.4 Mechanism of action of the combined therapy on CD

**Figure 3:**
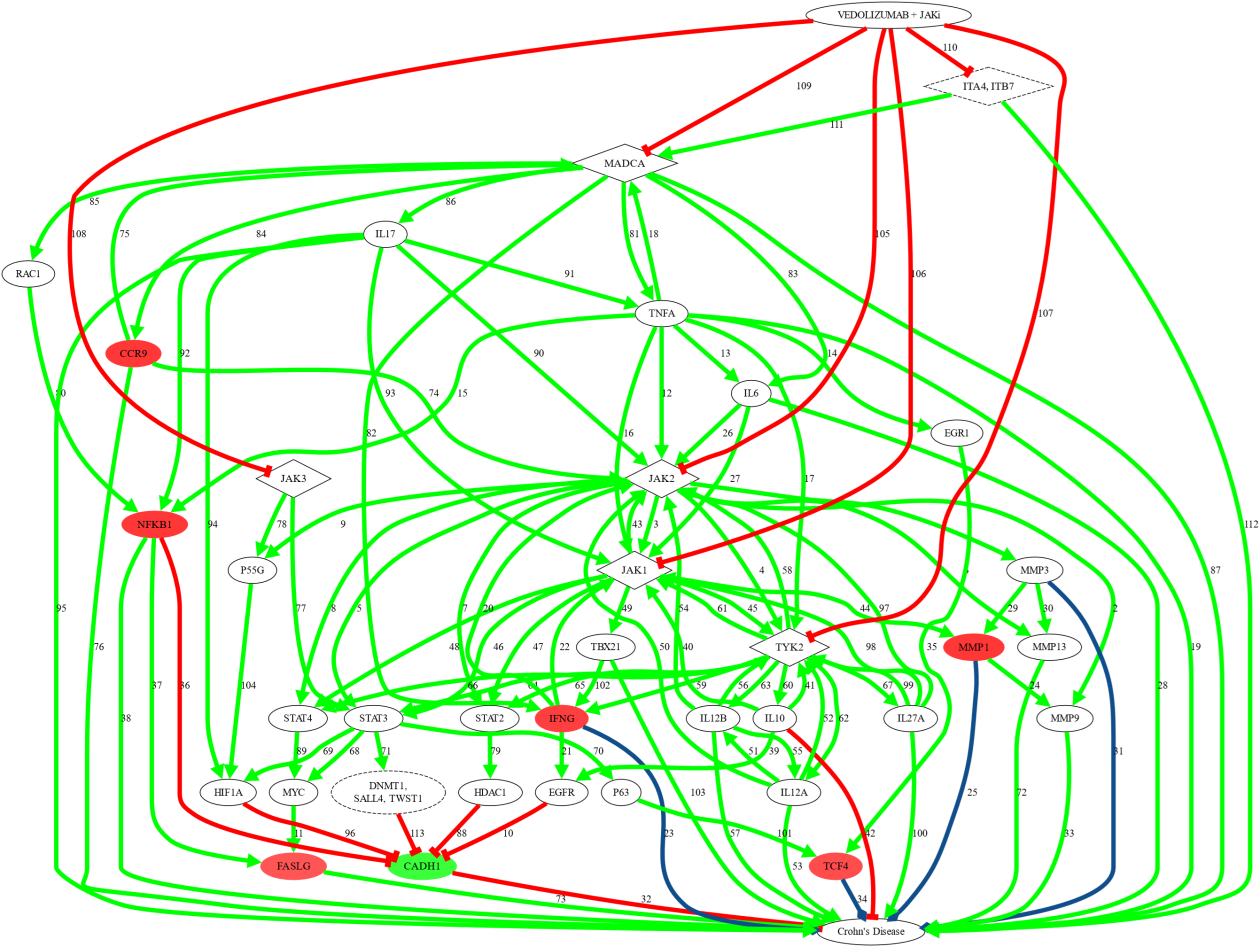
Predicted representation of the mechanism of action (MoA) of VDZ plus JAKi in CD. Figure created to represent MoA prediction for VDZ plus JAKi in CD using Graphviz software. All links have been manually reviewed: the numbers of the links correspond to the reference in **Supplementary Table S6**. Green arrows indicate activation; red lines indicate inhibition; blue lines indicate complex or dual relationships; rhombuses indicate drug targets; broken-lines indicate a node that contains more than one protein, all acting in the MoA together; full-filled circles indicate convergent effectors.

A representation of the predicted MoA of the combined therapy is graphically depicted in **Figure 3** and **Supplementary Figure 2**, with supporting references for the interactions provided in **Supplementary Table S6**.

The combined therapy seems to affect several processes implicated in motives M1, M3, and M4. Specifically, in M1, VDZ appears to mitigate intestinal barrier dysfunction by inhibiting TNF-α (inhibition of tight junction (TJ) dysfunction), while JAKi would act by decreasing metalloprotease activity, and both VDZ plus JAKi, when used together, by activation of CADH1 (restoration of TJ) and reduction of IFNG expression. Interestingly, VDZ might have an additive effect on JAKi’s mechanism of action reducing the production of the pro-inflammatory agents TNF-α, IFNG, IL17, and IL6, which normally promote the activation of their associated JAKs. Moreover, the damage to the intestinal architecture caused by local inflammation might be reversed by VDZ’s inhibition of TNF-α and NF-kB pathway, as well as by the inhibition of IFNG by both drugs.

The combined treatment may impact M3 through restriction of T cell gut-homing, diminution of Th1 cell activity in CD-affected tissue, and blockage of Th17 response. VDZ-mediated reduction in TNF-α expression might decrease NFKB1 activity and therefore FASLG expression. Regarding the diminution of Th1 cell activity in CD-affected tissue, JAKi’s effect in combination with VDZ-mediated reduction in TNF-α expression in the lamina propria could in turn further decrease Th1 activity in CD. The predicted MoA suggests that Th17 response might be blocked through the reduction of IL6, IFNG, and IL17 intestinal production by VDZ, resulting in reduced migration of effector T lymphocytes to the inflamed gut.

Finally, regarding M4, the combination therapy might reduce intestinal fibrosis by blocking extracellular matrix remodelling. This effect is achieved through the reduction of collagen synthesis (by VDZ through decrease of TNFA, IL17 and IL6 expression) and the inhibition of MMPs’ extracellular matrix (ECM) remodelling function (by JAKi via NF-κB p65 nuclear translocation inhibition and TCF4 expression reduction). Moreover, VDZ plus JAKi therapy might promote improvement in CD patients’ by decreasing stricture formation through the modulation of TNFA, IL17, IL6, NF-kB, and MMPs expression.

### 3.5 Mathematical modelling corroboration through bioflags analysis

The bioflags (as defined above) described in the literature for VDZ’s and JAKi’s actions on CD were evaluated in the single and combined treatment generated models to corroborate the simulate drug effects (see **Supplementary Table S5** for bioflags summary). Results of bioflag analysis are summarized in **Table 3**. From the 21 bioflags described for VDZ and included in both mathematical models (single drug and combined), 20 were optimally modulated to promote a healthy status in CD (**Table 3A**). Likewise, from the 14 bioflags described for JAKi and included in both mathematical models, 11 were correctly modulated in CD (**Table 3B**). Only bioflags from the characterisations of the drugs with higher evidence of modulation, other than just high-throughput data, were evaluated in our analysis. Overall, our models accurately represented the effects of VDZ and JAKi treatment observed and reported in patients according to scientific literature.

**Table 3:** Corroboration of the bioflags’ activation status after treatment with individual and combined treatment.

A				
Uniprot	Name	Bioflag sign	Vedolizumab	Vedolizumab+JAKi
P14902	IDO1	▼	✓	✓
P08047	SP1	▼		
P02751	FN1	▼	✓	✓
P19875	CXCL2	▼	✓	✓
P19876	CXCL3	▼	✓	✓
P25024	CXCR1	▼	✓	✓
P25025	CXCR2	▼	✓	✓
P32207	CXCR5	▼	✓	✓
P51677	CCR3	▼	✓	✓
P09919	CSF3	▼	✓	✓
Q99062	CSF3R	▼	✓	✓
P32927	CSF2RB	▼	✓	✓
P26748	NCF1	▼	✓	✓
P15153	RAC2	▼	✓	✓
O60603	TLR2	▼	✓	✓
Q9Y2C9	TLR6	▼	✓	✖
Q9HC29	NOD2	▼	✓	✓
P36222	CHI3L1	▼	✓	✓
Q16552	IL17A	▼	✓	✓
Q9NPF7	IL23A	▼	✓	✓
Q9HBE5	IL21R	▼	✓	✓
O43927	CXCL13	▼	✓	✓

B					
Uniprot	Name	Bioflag sign	JAKi	Vedolizumab+JAKi	
P15248	IL9	▼	✖	✓	*
Q14213	EBI3	▼	✖	✖	*
P36222	CHI3L1	▼	✓	✓	
P02461	COL3A1	▼	✓	✓	
P20718	GZMH	▼			
P19113	HDC	▼	✓	✓	
P13725	OSM	▼	✓	✓	
P18509	ADCYAP1	▼			
P05109	S100A8	▼	✓	✓	
Q9UL17	TBX21	▼	✓	✓	
P04216	THY1	▼			
P60568	IL2	▼	✓	✓	
P13232	IL7	▼	✓	✖	*
P40933	IL15	▼	✓	✓	
Q9HBE4	IL21	▼	✓	✓	
P40763	STAT3	▼	✓	✓	
P42224	STAT1	▼	✓	✓	

(A) The sign of VDZ bioflags is indicated together with the corroboration of the corresponding sign after VDZ and the combined treatment. (B) The sign of JAKi bioflags is indicated together with the corroboration of the corresponding sign after JAKi and the combined treatment. Red triangle indicates inhibition. Green checkmark corroborates and red cross antagonises the activation status in **“**Bioflag sing**”** column. Asterisks mark the proteins that had the defined (+/-) activation status in the model but did not pass the activation threshold (>|0.1|) defined by Anaxomics.

## 4 Discussion

CD presents a formidable challenge in the field of gastroenterology, characterised by its chronic, inflammatory, and potentially life-threatening course. Despite significant advancements, the aetiology of CD remains elusive, its incidence is increasing worldwide, and therapeutic options often fall short in providing long-term remission and relief for afflicted patients (1, 3, 4, 12). The results of the present work, by means of systems biology and AI, have provided the predicted MoAs that would herewith offer a mechanistic rationale to the use of VDZ plus JAKi as a promising therapy for CD patients. One of the central tenets of our investigation was the recognition of the progressive and destructive evolution of CD (35), which underscores the critical need for integrated therapeutic interventions more aligned with the multiple facets of CD pathophysiology. Our *in silico* modelling analysis reveals that combined effects of both drugs could optimally modulate a higher array of altered effectors in CD than each drug individually. Furthermore, both drugs converge in the modulation of several effectors though different pathways, which could potentiallyenhance their action and reinforce the beneficial effects observed in UC patients treated with this combined therapy (23). Importantly, recent real-world clinical experience also supported the potential of this dual targeted approach in CD (24). The results report the superior effect of VDZ compared to JAKi in the early stages of the disease (6 over 3 CD reverted effectors from M1), which reinforces the rational of an earlier use of VDZ to slow down the evolutionary process of the disease, according to purely immunological criteria. Moreover, the combined treatment distinguishes itself from others, by addressing distinct stages and immune sites on CD. Specifically, this combination targets both the initial, milder phase characterized by inflammatory conditions as well as the more advanced phase marked by complications, thus potentially offering potential benefits to CD patients. Hence, ours is the first study to describe the potential mechanisms of action underlying the beneficial effect of combined VDZ plus JAKi therapy in CD through systems biology techniques. By leveraging the complementary and convergent effects of both drugs, the combined therapy emerged as a compelling therapeutic strategy, since it holds the potential to achieve more comprehensive and sustained disease control in CD patients, particularly those with refractory or severe disease phenotypes. While VDZ acts by blocking immune cell trafficking to the intestinal submucosa, JAKi act locally at the inflammatory site. The combination of VDZ with JAKi targets two distinct stages of the disease, thereby providing potential advantages for CD patients.

Therefore, the combination of VDZ plus JAKi, unlike other combined treatments, would act at two different stages of the disease, thus offering potential greater benefits to CD patients.

The clinical course of IBD is highly heterogeneous and unpredictable, with multiple and serious complications that range from stricture formation to bowel obstruction or perforation, fistula formation and the need for surgery (36). CD development occurs in three stages´ (34, 37). The intestinal epithelium orchestrates the equilibrium between luminal content and mucosal immune system through its mechanical function as a physical barrier as well as its role in immune response (4). Penetration of bacteria and antigenic material into the bowel wall, facilitated by environmental factors and disruption of intestinal barrier would correlate with motive M1 of our study (intestinal barrier disruption). Secondly, an altered acute inflammatory response due to defective secretion of pro-inflammatory cytokines by macrophages and neutrophils, with impaired clearance of foreign material, which would reflect motive M2 of our study (defective innate immune response). Thirdly, these processes would lead to chronic inflammation and skewed adaptive Th1 and Th17 immune response, which would correlate with motive M3 of our analysis (chronic inflammation and predominant Th1/Th17 adaptive immune response). All these stages contribute to the characteristic features of the CD lesion, and to the subsequent tissue remodelling, which induces irreversible changes (motive M4 of our study). Nevertheless, further research would be required to fully establish the precise temporal sequence of CD progression and the physiological validity of the pathological processes (motives) identified in our study.

**Figure 4:**
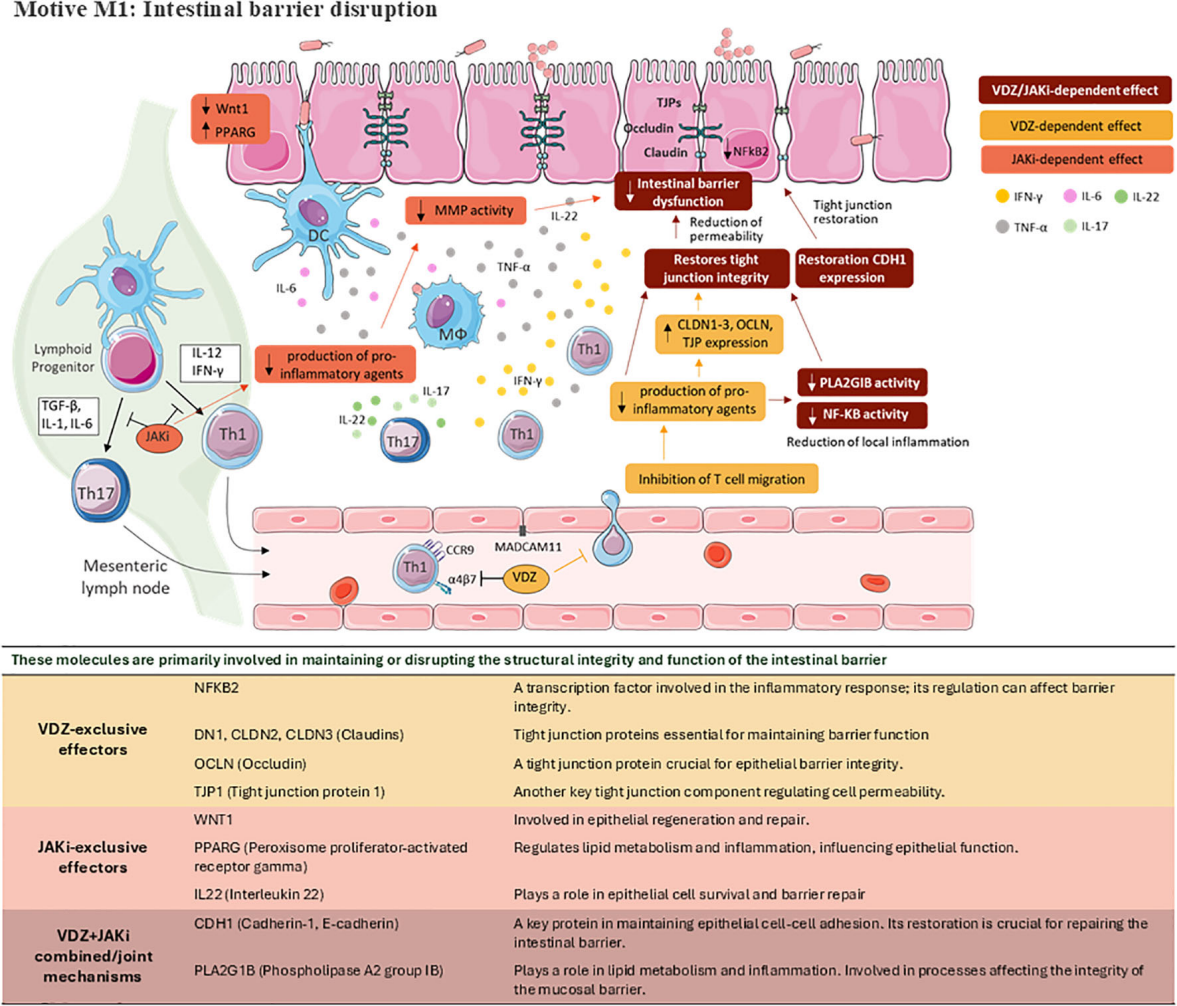
Schematic representation of key pathophysiological mechanisms modulated by VDZ, JAKi or its combination on CD. Motive M1: Intestinal barrier disruption. This figure was drawn using images from Servier Medical Art, which is licensed under CC BY 4.0 (https://creativecommons.org/licenses/by/4.0/).

**Figure 5:**
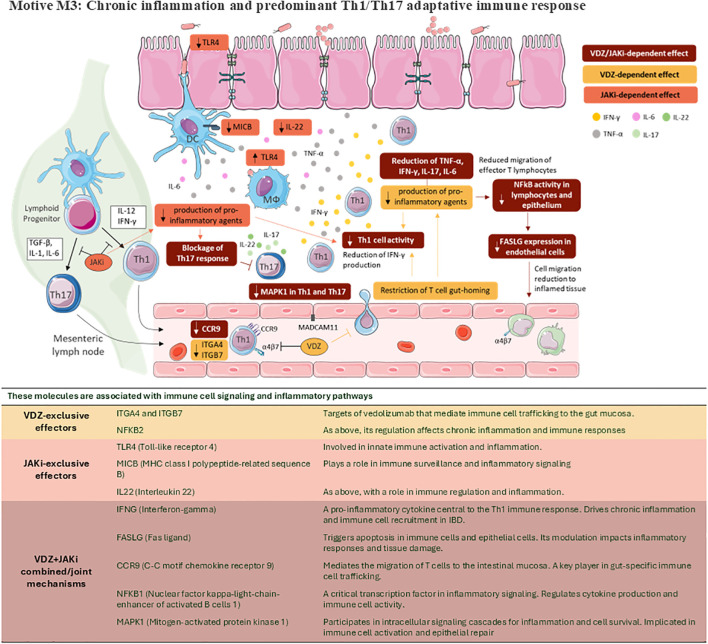
Schematic representation of key pathophysiological mechanisms modulated by VDZ, JAKi or its combination on CD. Motive M3: Chronic inflammation and predominant Th1/Th17 adaptative immune response. This figure was drawn using images from Servier Medical Art, which is licensed under CC BY 4.0 (https://creativecommons.org/licenses/by/4.0/).

**Figure 6:**
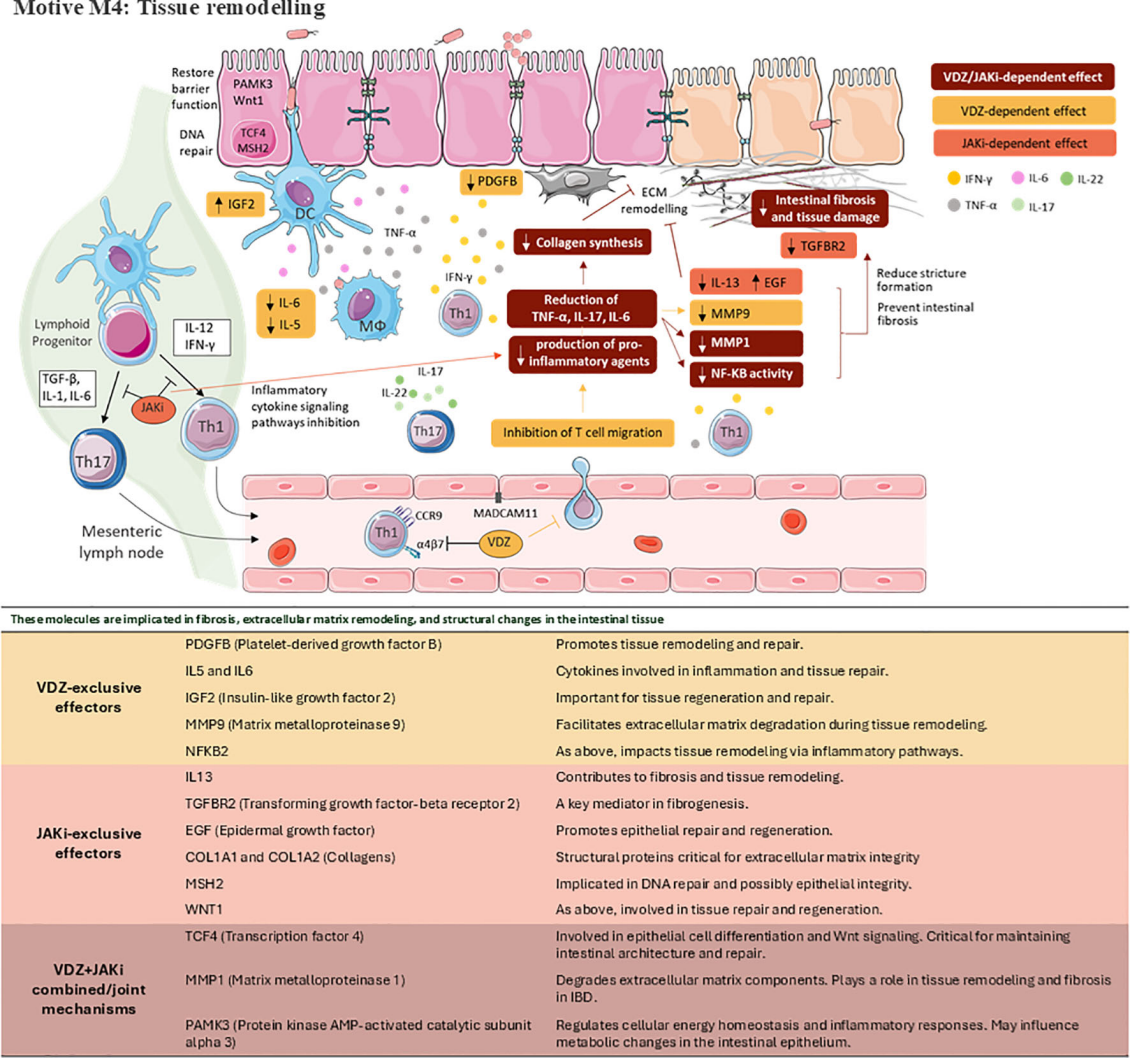
Schematic representation of key pathophysiological mechanisms modulated by VDZ, JAKi or its combination on CD. Motive M4: Tissue remodelling. This figure was drawn using images from Servier Medical Art, which is licensed under CC BY 4.0 (https://creativecommons.org/licenses/by/4.0/).

The rationale behind the combined action of VDZ and JAKi stems from their distinctive and complementary MoAs, with convergent effects on key effectors implicated in CD pathophysiology. According to the generated mathematical models, several molecular mechanisms would justify VDZ plus JAKi as an ideal combination therapy for CD. First, we explored the individual impact on CD pathophysiology from each drug. Regarding VDZ, a gene network analysis reported that VDZ-induced CD remission was associated with substantial effects on innate immune response, including changes in the expression of pattern recognition receptors, chemokines, and innate effector molecules (19). VDZ was specifically developed to block T-cell trafficking to the gut, an early stage process in the inflammatory cascade, but recent evidence suggests that this may not be its sole MoA (10, 16). Diverse effects have been proposed for VDZ action by binding to α4β7 integrin: alteration of blood monocytes gene expression, skewing the population toward a wound-healing and anti-inflammatory phenotype, inhibition of blood monocytes’ and DCs’ ability to enter intestinal epithelium (10, 16). Although different integrin interactions can facilitate pathological inflammation, only the α4β7 integrin-MAdCAM-1 interaction is selective for the gastrointestinal tract (8). In contrast, other treatments like JAKi work at later stages by targeting cytokine signalling, thereby reducing inflammation. In agreement with this, our model suggests that VDZ might induce the downregulation of proinflammatory mediators, such as TNF-α and IL6, secreted by T cells but also from monocytes and/or DCs. However, our models are multicellular, and the CD characterisation is not cell type-specific; therefore, we cannot differentiate between immune cell types. In parallel, JAKi represents a novel class of therapeutic agents that target JAK, key mediators of cytokine signalling pathways involved in immune regulation and inflammation (20, 38). By inhibiting JAK signalling, JAKi effectively modulates immune responses and cytokine production, thereby exerting potent anti-inflammatory effects (20, 39). Cytokines such as IL-6, IL-10, IL-2, or IL-22, dependent on JAK signalling, are essential for immune and stromal gut cell homeostasis (20, 38), and it has been described that JAKs are upregulated in active UC (20, 40). In our analysis, VDZ mainly modulates M3 (chronic inflammation and Th1/Th17 adaptive immune response), but as a consequence it also has an impact on M1 (intestinal barrier disruption) and M4 (tissue remodelling). On the other hand, JAKi mainly modulates M3 but, as a consequence, it also has an impact on M1 and M4 (**Figures 4**–**6**).

Second, we must consider the global impact of the combination therapy on CD pathophysiology. The combination of VDZ plus JAKi allows modulation of greater proportion of CD effectors (54.8%) than either drug alone, potentially offering enhanced therapeutic benefits for patients. Both drugs overlap in the modulation of several effectors (36%), which could probably enhance their combined therapeutic effects and increase efficacy, as observed for UC patients. Additionally, each drug provides complementary mechanisms that improve CD pathophysiology. Thus, 10% of the reversed effectors are given by the effects of VDZ and 9% by JAKi. The combination mainly affects M3, where both drugs show higher coincidence, although mechanistically they are different, thus each providing complementary benefits. The combination can also impact on M1 and M4. Although the mechanisms of convergence are less than in M3, in M4 it seems that there is a greater contribution or individual complementarity of each drug. Regarding M1, VDZ is the one that would provide more complementary mechanisms apart from those that already converge in both drugs.

### 4.1 Identified motives (key pathophysiological processes) detailed discussion

With regard to the M1 motive of our analysis (intestinal barrier disruption), we can make several considerations. Disruption of the intestinal barrier function is a hallmark feature of CD pathophysiology, facilitating the translocation of luminal antigens and bacteria into the underlying mucosa, thereby triggering inflammatory responses and perpetuating mucosal damage (2, 4, 7, 41, 42). In CD, there is an alteration of many components of the mucosal barrier, including death of intestinal epithelial cells, epithelial cell organization, impairment functions of epithelial cells, and complement activation, among others (2, 11, 41, 43, 44). Perturbations in barrier function in human IBD include reductions in barrier and antimicrobial secretions, reduced number of secretory cells, increased permeability, disabled tight junctions, through to substantial reduction, and even complete loss of the epithelium where ulceration occurs (42). VDZ seems to reduce intestinal barrier dysfunction by inhibiting TNF-α and both, VDZ and JAKi, by activation of CADH1, which is correlated with epithelial inflammation of the healthy mucosa adjacent to CD lesions (2, 45). For its part, JAKi might be able to reduce intestinal barrier dysfunction by decreasing metalloprotease activity (46). And both VDZ and JAKi treatments have an effect on the intestinal barrier dysfunction through the modulation of IFNγ expression (2, 47). Additionally, VDZ might have an additive effect on JAKi’s MoA due to its reducing effect on the production of pro-inflammatory mediators. The damage in the intestinal wall caused by local inflammation might be reversed by inhibition of INFγ, TNF-α and NF-kB (2, 48). VDZ-induced inhibition of integrin α4β7 binding to MAdCAM-1 very likely blocks recruiting T cells to the intestine, leading to chronic inflammation improvement via the reduction of proinflammatory molecules expression (TNFα, IFNγ, IL6 and IL17) in the gut (49, 50). Infiltration of blood monocytes into local tissues is facilitated through tight α4β7-MAdCAM-1 interactions, among other adhesion molecules and cadherin interactions (51). Approximately 5% of non-classical monocytes express α4β7 integrin, so it has been suggested that VDZ may actually disrupt intestinal wound healing and lead to complications (52). VDZ binds α4β7 integrin, which alters gene expression of blood monocytes, skewing the population towards a wound-healing and anti-inflammatory phenotype. Also, VDZ binds to α4β7 integrin on pro-inflammatory blood monocytes, thereby inhibiting their ability to enter the intestinal epithelium (10). Finally, VDZ-mediated TNF-α reduced expression might reduce NFKB1 and therefore FASLG expression in endothelial cells, which would impede increased cell migration to the inflamed tissue (**Figure 4**) (53, 54).

Central to the pathogenesis of CD is the dysregulation of immune responses within the intestinal mucosa, leading to chronic inflammation and tissue damage. Both innate and adaptive immune mechanisms contribute to the perpetuation of inflammation in CD, with dysregulated cytokine signalling pathways playing a central role (55, 56). Regarding the motive M3 of our study (chronic inflammation and Th1/Th17 adaptive immune response), both Th1 and Th17 cytokines are upregulated in CD (57). Many actions previously ascribed to the Th1 cytokine IL12 may have been mediated by IL23, an inducer of Th17 cells, since IL12/IL23p40 is part of both cytokines (57). CD-associated inflammation is supposed to be driven by Th1/Th17 cell-derived cytokines, even though there is evidence that the mucosal profile of cytokines may vary with the evolution of the disease (55). CD pathophysiology evolves as the disease progresses through separate but interrelated phases; early CD (phase I) is characterised by a polarized Th1-type response, whereas late CD (phases II & III) shows a strong Th2 response. Treatments targeting mechanisms in early disease stages, such as VDZ, may be more efficient in phase I before transitioning into the more advanced phases of CD. The early stage of CD inflammation is dominated by Th1 cytokines, while a mixed Th1/Th17 response is seen in areas with early or established lesions (55). The imbalance of proinflammatory and anti-inflammatory cytokines, activated leukocyte infiltrations, and increased expression of adhesion molecules and chemokines in intestinal mucosa further promote immunopathological damage in the gut mucosa of IBD (56). According to the predicted mechanism of action, JAKi could reduce Th1 cell activity by inhibiting Th1-cell production of IFNG, acting on IL12 producing JAK+ cells located in the lamina propia (2). The combination of JAKi’s effect with VDZ-mediated TNFA reduced expression in the lamina propria could enhance the diminution of Th1 activity in CD (48). The combination therapy could have a great impact on Th17 response blockage, which plays a pivotal role in CD (**Figure 5**) (1, 7, 58).

In addition to chronic inflammation and intestinal barrier dysfunction, tissue remodelling represents another key aspect of CD pathogenesis. The prototypical and most common type of tissue remodelling in IBD, and CD in particular, is a fibrotic response (36, 59). With respect to motive M4 of our study (tissue remodelling), chronic inflammation within the intestinal mucosa leads to fibrosis and tissue damage, which can culminate in the development of complications such as scarring. In a hollow muscular organ, this scarring causes narrowing, stricture formation, fistulas, and abscesses, which might result in surgery (7, 36, 60). Based on its clinical behaviour, CD is classified as B1 (non-stricturing, non-penetrating), B2 (stricturing) and B3 (penetrating) (60). There are several factors involved in tissue remodelling and extracellular matrix remodelling events, including the site and duration of inflammation, soluble molecules, the gut microbiota, and the type of mesenchymal cell response (36). The predicted MoA from our study suggests that VDZ might decrease TNF-α, IL17 and IL6 expression, leading to a reduced collagen synthesis while JAKi, probably via NF-κB p65 nuclear translocation inhibition and TCF4 expression reduction, might be able to inhibit MMPs expression, including MMP9, which is the predominant upregulated remodelling protease in inflamed intestinal tissue (61). The combination therapy might reduce intestinal fibrosis through the reduction of collagen synthesis and MMPs’ ECM remodelling function. VDZ, primarily targeting T-cell trafficking to the gut and inflammatory activity in early disease stages, may indirectly prevent further damage, while JAKi, by inhibiting inflammatory cytokine signalling pathways, directly impact collagen synthesis and MMP-mediated ECM remodelling. This dual approach could prevent ulterior damage, since most patients with CD will eventually develop structuring or perforating complications (35). Future clinical trials would be needed to answer this question. Moreover, the inhibition of collagen synthesis and MMPs’ ECM remodelling function by the combined therapy would also provide a benefit over fibrosis-promoted stricture formation. VDZ plus JAKi therapy might promote CD patients’ improvement by decreasing stricture formation through TNF-α, IL17, IL6, NF-kB, and MMPs expression modulation (**Figure 6**) (36).

### 4.2 Study limitations, strengths, future prospects, and final conclusions

Previous studies performed in other clinical conditions demonstrated the potential translational use of TPMS technology (27, 28, 30). However, this *in silico* modelling approach and its biological validation are limited by the information about disease, drugs, and available data in public repositories. Moreover, the models’ evaluations are also limited by the publicly available interactome information. Despite these limitations, the methodology employed for the modelling, considering the whole human protein network and a wide range of drug-pathology functional information (either as indication or adverse events), allow the TPMS-based model to infer biological and functional information connecting a wider biological spectrum. Our analysis was not only limited to the studied disease, which maximizes the applicability of our models for rare settings, such as exploration of mechanisms behind adverse events. Therefore, although the models and conclusions in our study could be updated over time as new information is generated (improving accuracy and allowing exploring unanswered questions), our approach provided results that are in agreement with current molecular, pathophysiological, and clinical knowledge as it is shown by the results on the corroboration of known bioflags for each drug. In the current study, the generated models presented accuracies against the training set of 93.95%. The mechanistic nature of the models results in a normalised predicted protein activity value for each protein that combines a variety of biological signals, from gene expression to protein regulation, signal transduction or even, degradation regulation, among others (30). This approach allows for the study of the convergence of different stimuli, in this case, VDZ plus JAKi. However, this complexity also requires a careful evaluation for *in vitro* or *in vivo* validation, in order to check the protein activity regulation at the correct level, either it is gene expression, protein translation, protein regulation, or so on. Nevertheless, corroboration of our findings will require well-designed controlled clinical trials in order to assess the real benefits of the analysed combined therapy in CD patients. It has to be taken into account that the objective of the application of this methodology was to obtain a more profound understanding of the molecular basis of the combined mechanisms of action of VDZ plus JAKi, while a direct correlation to specific clinical outcomes was not an intended aim of the study. Recent real-world clinical data already support the efficacy and safety of this combination (24). In addition,in this context, the VICTRIVA Study will provide evidence-based foundation for dual targeted therapy with VDZ plus JAKi (upadacitinib) in moderate to severe CD (62). However, clinically relevant measurements of some of the presented molecular findings have already been found to correlate to features of remission: serum or fecal MMP-9 correlates with disease activity, fecal calprotectin and endoscopic findings (63–65); serum IL-17, which is a key player in Th17 response, has been suggested as a comparable endoscopic marker to the high-sensitivity CRP assay (66); and serum markers of collagen metabolism, regulated by several of the proteins highlighted in this study, have been found to correlate to both tissular damage (PRO-C6) and inflammatory changes (C4M and PRO-C4) (67). Measurement of these molecules, as well as their histological and clinical correlates, could be used to corroborate the convergent mechanisms described in this study.

It would also be of great interest to apply our *in silico* analysis to UC, constructing specific models for this disease. Comparison between UC and CD would allow identifying other proteins or pathways of interest that have not been identified in the present study. Combined therapy might be a treatment option, mainly in IBD patients with refractory disease (3, 9). Our technology can shed some light on this aspect, but large-scale efficacy and safety studies would be needed to confirm the benefits of VDZ plus JAKi combined therapy.

Another valuable prospective application of this approach would be to construct interactomes for inadequate versus adequate responders to vedolizumab and JAKi, integrating molecular or biomarker data (e.g., transcriptomic or proteomic profiles) together with curated mechanistic information on non-response. The ensemble nature of the TPMS framework, which generates multiple plausible molecular configurations, could help represent this variability, even considering inter-patient heterogeneity arising from pathophysiological processes like chronic inflammation, Th1/Th17 immune deregulation (M3), or intestinal tissue remodeling (M4), which likely vary among long-term moderate or severe CD patients. Although a specific analysis of these aspects was beyond the scope of the present work, extending the models with new patient data or theoretical assumptions could thus reveal how vedolizumab and JAKi act across disease stages or degrees of dysregulation, potentially clarifying mechanisms underlying differential or complementary responses. Similar applications of this methodology have explored steroid response in ulcerative colitis (28), suggested characteristics of theoretical responder ranges to certolizumab in psoriasis models (68), and the hypothesized markers for early cellular damage in Fabry disease (69) and skeletal complications in Gaucher disease (70). In summary, the culmination of our project has unveiled a promising avenue in the field of CD treatment: the potential combined effects of VDZ plus JAKi. Through a comprehensive and integrated *in silico* analysis of CD pathophysiology, and a meticulous execution, we have uncovered the underlying mechanisms of action and offered the mechanistic rationale that underpins the viability of combining these two therapies. Moreover, by modulating a significantly higher percentage (54.8%) of effectors compared to individual drug treatments, this combined approach holds the promise of delivering greater therapeutic benefits to patients. What is particularly intriguing is the convergence of effectors modulated by both VDZ and JAKi, accounting for 36% of the total. This overlap suggests a potential synergistic enhancement of their respective effects when used in tandem, mirroring the observed efficacy in UC patients who have undergone combination therapy (23). Additionally, this drug combination might be beneficial for non-responder patients to current treatments for CD, as supported by recent clinical experience in refractory CD patients treated with VDZ and a JAKi (24). Therefore, by elucidating the putative synergistic effects of VDZ and JAKi in modulating diverse pathophysiological processes implicated in CD, our findings pave the way for the development of more effective and personalised treatment strategies for this debilitating condition.

## Data Availability

The original contributions presented in the study are included in the article/**Supplementary Material**. Further inquiries can be directed to the corresponding author.

## Supplementary material

Supplementary Figure 1 Preliminary Signal of VDZ or JAKi on CD and on each pathophysiological motive. Preliminary models of each drug individually were made to determine the effects of VDZ and JAKi on each identified CD motive and on whole CD physiopathology. None of the treatments seem to have impact on motive M2 (defective innate immune response).

Supplementary Figure 2 Simplified version of **Figure 3**. Predicted representation of the mechanism of action (MoA) of vedolizumab (VDZ) plus JAKi in Crohn’s disease (CD). This revised figure provides a more streamlined and interpretable visualization of the model by removing redundancies and grouping proteins with shared functions in the context of the MoA. It additionally includes information on the pathophysiological motives modulated by each pathway: **(A)** M1 - Intestinal barrier disruption; **(B)** M3 - Chronic inflammation and Th1/Th17 adaptive immune response; and **(C)** M4 - Tissue remodelling. The figure was generated using Graphviz software to represent the predicted MoA of VDZ plus JAKi in CD. All links have been manually reviewed, and the corresponding reference numbers for each link can be found in the original **Figure 3** and in **Supplementary Table S6**. Green arrows indicate activation; red lines indicate inhibition; rhombuses indicate drug targets; broken lines indicate nodes containing more than one protein acting jointly in the MoA; and filled circles indicate convergent effectors.
