# Supplementary figures and images for: Synergistic effects of vedolizumab and JAK 1,2,3 inhibitors in Crohn’s disease: insights from a systems biology and artificial intelligence-based approach
Source: Front Immunol. 2025 Dec 10;16:1699203. doi: 10.3389/fimmu.2025.1699203 (PMC12728006; doi:10.3389/fimmu.2025.1699203)

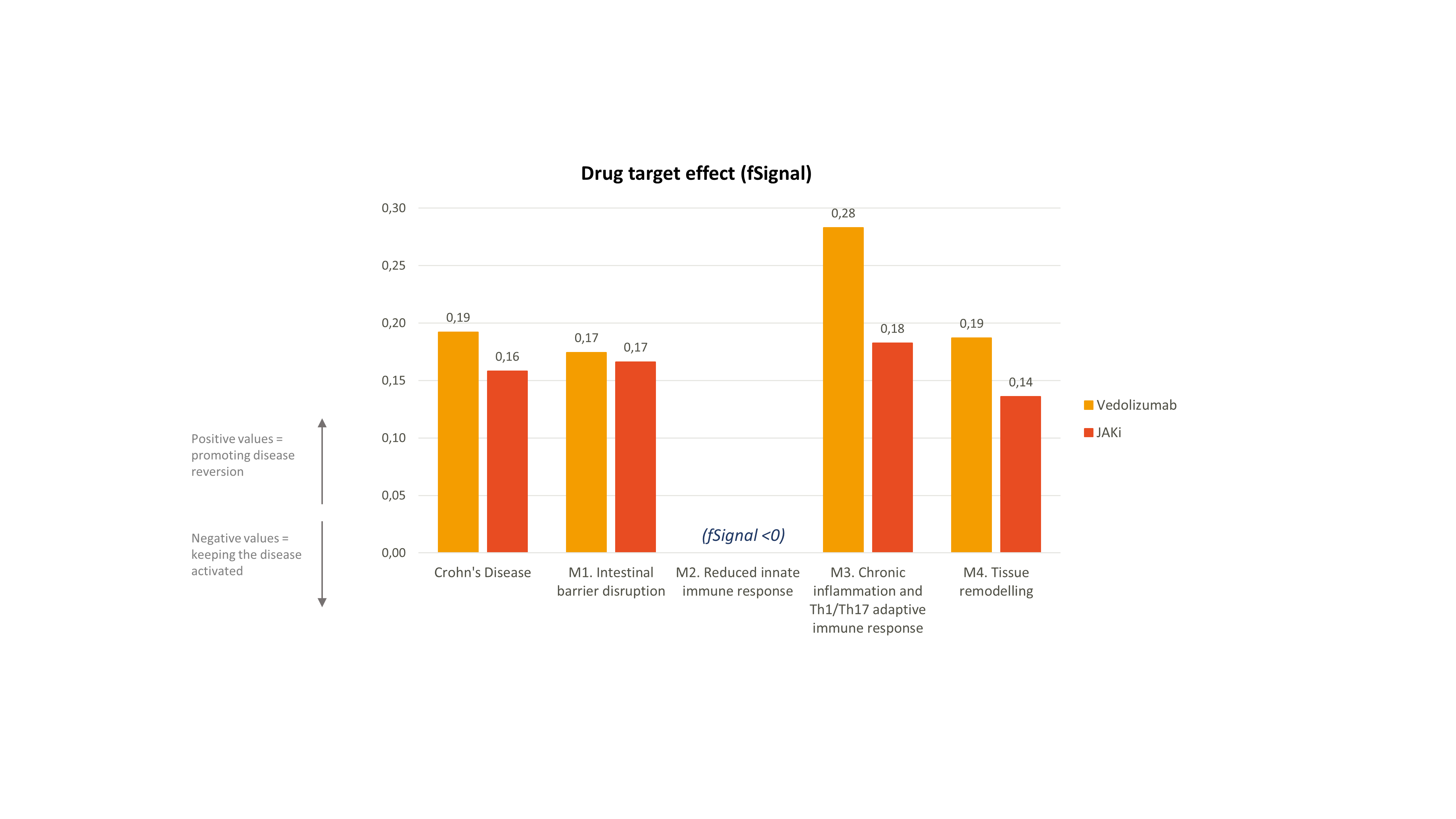

Supplement: Supplementary Figure 1 — Preliminary Signal of VDZ or JAKi on CD and on each pathophysiological motive. Preliminary models of each drug individually were made to determine the effects of VDZ and JAKi on each identified CD motive and on whole CD physiopathology. None of the treatments seem to have impact on motive M2 (defective innate immune response). [file Image1.tif]

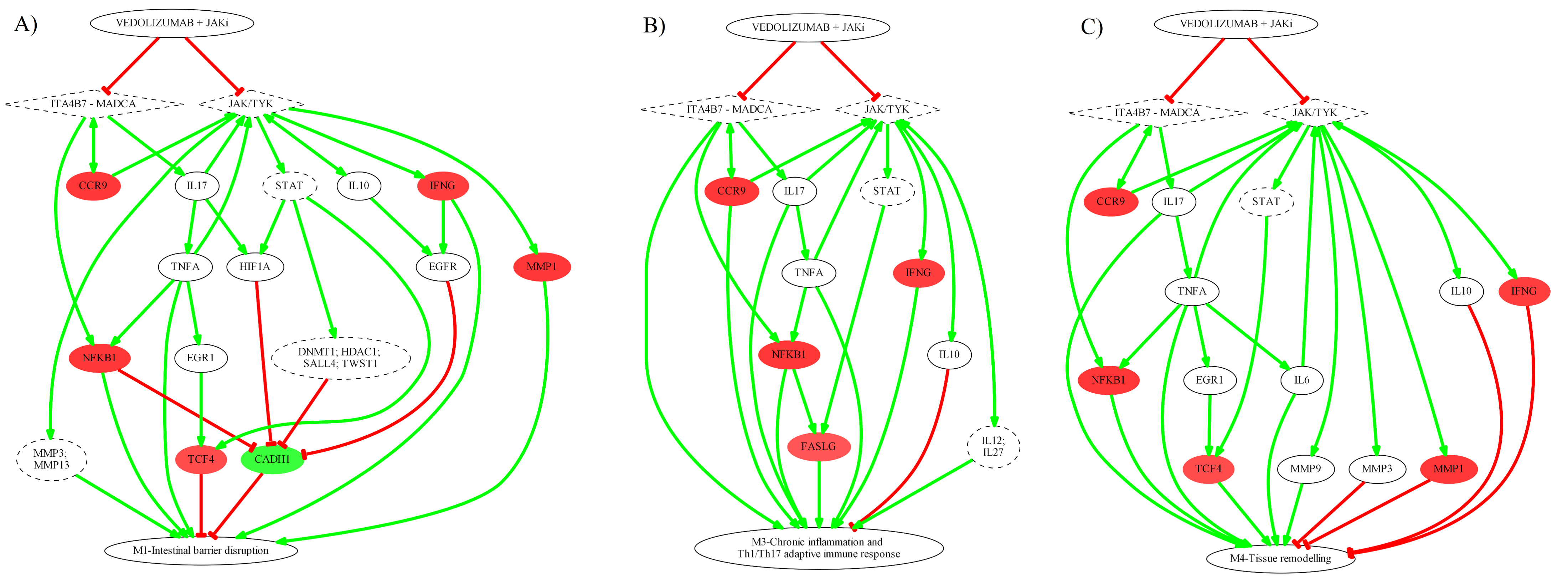

Supplement: Supplementary Figure 2 — Simplified version of Figure 3. Predicted representation of the mechanism of action (MoA) of vedolizumab (VDZ) plus JAKi in Crohn’s disease (CD). This revised figure provides a more streamlined and interpretable visualization of the model by removing redundancies and grouping proteins with shared functions in the context of the MoA. It additionally includes information on the pathophysiological motives modulated by each pathway: (A) M1 - Intestinal barrier disruption; (B) M3 - Chronic inflammation and Th1/Th17 adaptive immune response; and (C) M4 - Tissue remodelling. The figure was generated using Graphviz software to represent the predicted MoA of VDZ plus JAKi in CD. All links have been manually reviewed, and the corresponding reference numbers for each link can be found in the original Figure 3 and in Supplementary Table S6. Green arrows indicate activation; red lines indicate inhibition; rhombuses indicate drug targets; broken lines indicate nodes containing more than one protein acting jointly in the MoA; and filled circles indicate convergent effectors. [file Image2.tif]
