# Supplementary material for: Synergistic effects of vedolizumab and JAK 1,2,3 inhibitors in Crohn’s disease: insights from a systems biology and artificial intelligence-based approach
Source: Front Immunol. 2025 Dec 10;16:1699203. doi: 10.3389/fimmu.2025.1699203 (PMC12728006; doi:10.3389/fimmu.2025.1699203)
Supplement: Supplementary file 3 [file Table1.docx]

Title: Synergistic effects of vedolizumab and JAK 1,2,3 inhibitors in Crohn's disease: insights from a systems biology and artificial intelligence-based approach

Authors: Ignacio Marín-Jiménez, Mónica Sierra-Ausín, Teresa Letosa-Abián, Jesús Aparicio, Carmen Montoto-Otero, Silvia Sánchez-Ramón.

Supplementary Table 1. Search strings in PubMed

| Search strings in PubMed | |
| --- | --- |
| CD pathophysiology | ("Crohn’s disease"[Title] OR "Crohn disease"[Title]) AND (pathogenesis[Title/Abstract] OR pathophysiology[Title/Abstract] OR molecular[Title/Abstract]) AND Review[ptyp] |
| VDZ MoA | ("vedolizumab"[Title] OR "VDZ"[Title] OR "LDP 02"[Title] OR "LDP-02"[Title] OR "LDP02"[Title] OR "MLN-0002"[Title] OR "MLN-02"[Title] OR "MLN0002"[Title] OR "MLN02"[Title] OR "entyvio"[Title]) AND ("crohn"[Title] OR "Crohn's disease"[Title] OR "Crohn Disease"[Title]) |
| JAKi MoA | ("JAKi"[Title] OR "JAK inhibitor"[Title] OR "Tofacitinib"[Title] OR "Xeljanz"[Title] OR "Tasocitinib"[Title] OR "Tofacitinibum"[Title] OR "CP 690550"[Title] OR "CP- 690 550"[Title] OR "CP- 690 free base"[Title] OR "CP-690-550"[Title] OR "CP-690,550"[Title] OR "CP-690,550 free base"[Title] OR "CP-690550"[Title] OR "CP-690550 free base"[Title] OR "CP690,550"[Title] OR "CP690550"[Title]) AND (("crohn"[Title] OR "Crohn's disease"[Title] OR "Crohn Disease"[Title] OR "CD") OR ("IBD" OR "Intestinal Bowel Disease") AND ("gene expression")); ("JAKi"[Title] OR "JAK inhibitor"[Title] OR "Upadacitinib"[Title] OR "Rinvoq"[Title] OR "ABT 494"[Title] OR "ABT-494"[Title]) AND (("crohn"[Title] OR "Crohn's disease"[Title] OR "Crohn Disease"[Title] OR "CD") OR ("IBD" OR "Intestinal Bowel Disease") AND ("gene expression")); (Filgotinib) AND (("crohn"[Title] OR "Crohn's disease"[Title] OR "Crohn Disease"[Title] OR "CD") OR ("IBD" OR "Intestinal Bowel Disease")) AND ("gene expression") |
